# How long do nosocomial pathogens persist on inanimate surfaces? A systematic review

**DOI:** 10.1186/1471-2334-6-130

**Published:** 2006-08-16

**Authors:** Axel Kramer, Ingeborg Schwebke, Günter Kampf

**Affiliations:** 1 Institut für Hygiene und Umweltmedizin, Ernst-Moritz-Arndt Universität, Greifswald, Germany; 2 Robert-Koch Institut, Berlin, Germany; 3 Bode Chemie GmbH & Co. KG, Scientific Affairs, Hamburg, Germany

## Abstract

**Background:**

Inanimate surfaces have often been described as the source for outbreaks of nosocomial infections. The aim of this review is to summarize data on the persistence of different nosocomial pathogens on inanimate surfaces.

**Methods:**

The literature was systematically reviewed in MedLine without language restrictions. In addition, cited articles in a report were assessed and standard textbooks on the topic were reviewed. All reports with experimental evidence on the duration of persistence of a nosocomial pathogen on any type of surface were included.

**Results:**

Most gram-positive bacteria, such as Enterococcus spp. (including VRE), *Staphylococcus aureus *(including MRSA), or *Streptococcus pyogenes*, survive for months on dry surfaces. Many gram-negative species, such as Acinetobacter spp., *Escherichia coli*, Klebsiella spp., *Pseudomonas aeruginosa*, *Serratia marcescens*, or Shigella spp., can also survive for months. A few others, such as *Bordetella pertussis*, *Haemophilus influenzae*, *Proteus vulgaris*, or *Vibrio cholerae*, however, persist only for days. Mycobacteria, including *Mycobacterium tuberculosis*, and spore-forming bacteria, including *Clostridium difficile*, can also survive for months on surfaces. *Candida albicans *as the most important nosocomial fungal pathogen can survive up to 4 months on surfaces. Persistence of other yeasts, such as *Torulopsis glabrata*, was described to be similar (5 months) or shorter (*Candida parapsilosis*, 14 days). Most viruses from the respiratory tract, such as *corona*, *coxsackie*, *influenza*, *SARS *or *rhino *virus, can persist on surfaces for a few days. Viruses from the gastrointestinal tract, such as *astro*virus, *HAV*, *polio- *or *rota *virus, persist for approximately 2 months. Blood-borne viruses, such as HBV or HIV, can persist for more than one week. Herpes viruses, such as CMV or HSV type 1 and 2, have been shown to persist from only a few hours up to 7 days.

**Conclusion:**

The most common nosocomial pathogens may well survive or persist on surfaces for months and can thereby be a continuous source of transmission if no regular preventive surface disinfection is performed.

## Background

Within the global infection control community, there is an ongoing controversy about the appropriate treatment of inanimate surfaces in hospitals in order to prevent transmission of nosocomial pathogens within an institution. Based on a lack of epidemiological data that would provide evidence of a benefit for the patient from surface disinfection (e.g., from a significant reduction of nosocomial infection rates), some scientists postulate that cleaning of surfaces with non-antimicrobial detergents is generally sufficient [1]. Others prefer cleaning of surfaces with antimicrobial agents, based on data on the risk of infection due to microbial contamination and potential transmission of nosocomial pathogens, at least in the immediate vicinity of patients [2-4].

New guidelines on treatment of surfaces in hospitals take into account more parameters which are considered to be relevant for preventing the transmission of nosocomial pathogens, such as the type of ward or the expected frequency of hand contact with a surface [5,6]. Irrespective of the divergent opinions regarding the appropriate treatment of surfaces, an important parameter for a fair scientific assessment remains, that is, the persistence of nosocomial pathogens on surfaces. The longer a nosocomial pathogen persists on a surface, the longer it may be a source of transmission and thus endanger a susceptible patient or healthcare worker. The aim of this review was therefore to collect and assess the data that have been published in the last decades on persistence of all types of nosocomial pathogens on surfaces, both in the context of surface disinfection and the control of nosocomial outbreaks.

## Methods

### Search strategy

The literature was systematically reviewed in MedLine on the internet homepage of the National Library of Medicine without language restrictions. The search was done on 29 December 2005 and covered all years available in MedLine. The following search terms were applied: persistence, survival, surface, fomite, bacteria, virus, pathogen, transmission, and nosocomial. In addition, the citations in each study found during the main search were reviewed for potential relevance. Finally, standard textbooks on infection control, bacteriology and virology were examined for information.

### Selecting studies

All reports with experimental evidence on the duration of persistence of a nosocomial pathogen on any type of inanimate surface were included. Information from textbooks was also included, even if the chapter itself did not contain experimental evidence. At least two of the investigators decided on the relevance of each report. Reports were not blinded to the investigators so that they knew the names of the authors of all studies.

### Interpretation of studies

For a clinically relevant summary, all nosocomial pathogens were grouped according to their importance in causing hospital-acquired hand-transmitted infections [7] and according to their mode of nosocomial transmission [8]. The range of the reported duration of persistence was used as the principle outcome of the search for each nosocomial pathogen. In addition, parameters with potential influence on persistence were evaluated in all experimental studies.

## Results

### Persistence of bacteria

Most gram-positive bacteria, such as *Enterococcus *spp. (including VRE), *Staphylococcus aureus *(including MRSA), or *Streptococcus pyogenes *survive for months on dry surfaces (Table 1). In general, there was no obvious difference in survival between multiresistant and susceptible strains of *Staphylococcus aureus *and *Enterococcus *spp. [9]. Only in one study was such a difference suggested, but the susceptible strains revealed a very brief survival as such [10]. Many gram-negative species, such as *Acinetobacter *spp., *Escherichia coli*, Klebsiella spp., *Pseudomonas aeruginosa*, *Serratia marcescens*, or *Shigella *spp. can survive on inanimate surfaces even for months. These species are found among the most frequent isolates from patients with nosocomial infections [11]. A few others, such as *Bordetella pertussis*, *Haemophilus influenzae*, *Proteus vulgaris*, or *Vibrio cholerae*, however, persist only for days (Table 1). Mycobacteria – including *Mycobacterium tuberculosis *and spore-forming bacteria, including *Clostridium difficile *– can also survive for many months on surfaces (Table 1).

**Table 1:** Persistence of clinically relevant bacteria on dry inanimate surfaces.

Type of bacterium	Duration of persistence (range)	Reference(s)
Acinetobacter spp.	3 days to 5 months	[18, 25, 28, 29, 87, 88]
*Bordetella pertussis*	3 – 5 days	[89, 90]
*Campylobacter jejuni*	up to 6 days	[91]
*Clostridium difficile *(spores)	5 months	[92–94]
*Chlamydia pneumoniae*, *C. trachomatis*	≤ 30 hours	[14, 95]
*Chlamydia psittaci*	15 days	[90]
*Corynebacterium diphtheriae*	7 days – 6 months	[90, 96]
*Corynebacterium pseudotuberculosis*	1–8 days	[21]
*Escherichia coli*	1.5 hours – 16 months	[12, 16, 17, 22, 28, 52, 90, 97–99]
Enterococcus spp. including VRE and VSE	5 days – 4 months	[9, 26, 28, 100, 101]
*Haemophilus influenzae*	12 days	[90]
*Helicobacter pylori*	≤ 90 minutes	[23]
Klebsiella spp.	2 hours to > 30 months	[12, 16, 28, 52, 90]
Listeria spp.	1 day – months	[15, 90, 102]
*Mycobacterium bovis*	> 2 months	[13, 90]
*Mycobacterium tuberculosis*	1 day – 4 months	[30, 90]
*Neisseria gonorrhoeae*	1 – 3 days	[24, 27, 90]
*Proteus vulgaris*	1 – 2 days	[90]
*Pseudomonas aeruginosa*	6 hours – 16 months; on dry floor: 5 weeks	[12, 16, 28, 52, 99, 103, 104]
*Salmonella typhi*	6 hours – 4 weeks	[90]
*Salmonella typhimurium*	10 days – 4.2 years	[15, 90, 105]
Salmonella spp.	1 day	[52]
*Serratia marcescens*	3 days – 2 months; on dry floor: 5 weeks	[12, 90]
Shigella spp.	2 days – 5 months	[90, 106, 107]
*Staphylococcus aureus*, including MRSA	7 days – 7 months	[9, 10, 16, 52, 99, 108]
*Streptococcus pneumoniae*	1 – 20 days	[90]
*Streptococcus pyogenes*	3 days – 6.5 months	[90]
*Vibrio cholerae*	1 – 7 days	[90, 109]

Overall, gram-negative bacteria have been described to persist longer than gram-positive bacteria [12,13]. Humid conditions improved persistence for most types of bacteria, such as *Chlamydia trachomatis *[14], *Listeria monocytogenes *[15], *Salmonella typhimurium *[15], *Pseudomonas aeruginosa *[16], *Escherichia coli *[17], or other relevant pathogens [18,19]. Only *Staphylococcus aureus *was found to persist longer at low humidity [16].

Low temperatures, e.g., 4°C or 6°C, also improved persistence of most types of bacteria, such *Listeria monocytogenes *[15], *Salmonella typhimurium *[15], MRSA [20], corynebacteria [21], *Escherichia coli *[17,22], *Helicobacter pylori *[23], and *Neisseria gonorrhoeae *[24].

The type of test material does not reveal a consistent result. Although some investigators report that the type of material has no influence on the persistence [25,26], other authors described a longer persistence on plastic [27,28], and others yet see a survival advantage on steel [29].

Other factors were rarely investigated and hence provide inconsistent results. Longer persistence has been described with higher inocula [28], in the presence of protein [13], serum [13,24], sputum [30], or without dust [10].

### Persistence of fungi

*Candida albicans *as the most important nosocomial fungal pathogen can survive up to 4 months on surfaces (Table 2). Persistence of other yeasts was described to be similar (*Torulopsis glabrata *5 months) or shorter (*Candida parapsilosis *14 days).

**Table 2:** Persistence of clinically relevant fungi on dry inanimate surfaces.

Type of fungus	Duration of persistence (range)	Reference(s)
*Candida albicans*	1 – 120 days	[31, 53, 99, 110]
*Candida parapsilosis*	14 days	[110]
*Torulopsis glabrata*	102 – 150 days	[31]

The presence of serum or albumin, a low temperature, and high humidity have been described as leading to longer persistence [31].

### Persistence of viruses

Most viruses from the respiratory tract such as *corona-*, *coxsackie-*, *influenza*virus, *SARS*, or *rhino*virus can persist on surfaces for a few days. Viruses from the gastrointestinal tract, such as *astro*virus, *HAV*, *polio- *and *rota*virus persist for approximately 2 months. Blood-borne viruses, such as HBV or HIV, can persist for more than one week. Herpes viruses such as CMV or HSV type 1 and 2 have been shown to persist from only a few hours up to 7 days.

The influence of humidity on persistence has been described inconsistently. For entero- [32] and *rhino*virus [33], high humidity was associated with longer persistence. *HSV *[34] and *HAV *[35] can persist longer at low humidity. For *aden*o- [32,34], *rota- *[36,37], and *polio*virus [34,35], conflicting results were reported.

For most viruses, such as *astro- *[38], *adeno- *[34], *polio*virus [34], *HSV *[34], and *HAV *[35], low temperature is associated with a longer persistence.

Inconsistent results are also reported for the influence of type of material. Some authors described that the type of material did not affect the persistence of *echo- *[39], *adeno- *[39-41], *parainfluenza- *[39], *rota*virus [41], RSV [39], *polio- *[41] or *noro*virus [42]. Other investigators found that persistence was favored on non-porous surfaces for *influenza*virus [43], on formica and gloves for *RSV *[44], and on a telephone receiver for *FCV *[45].

Other parameters for a longer persistence of viruses include the presence of fecal suspension [38] and a higher inoculum [46].

### Persistence of other nosocomial pathogens

Cryptosporidium species have been reported to survive on dry surfaces for only 2 hours [47].

## Discussion

The most relevant nosocomial pathogens can persist on dry inanimate surfaces for months. In addition to the duration of persistence, some studies have also identified factors influencing persistence. A low temperature, such as 4°C or 6°C, was associated with longer persistence for most bacteria, fungi and viruses. High humidity (e.g., > 70%) was also associated with longer persistence for most bacteria, fungi, and viruses, although for some viruses conflicting results were reported. A few studies also suggest that a higher inoculum is associated with longer persistence. The type of surface material and the type of suspension medium, however, reveal inconsistent data. Overall, a high inoculum of the nosocomial pathogen in a cold room with high relative humidity will have the best chance for long persistence.

In most reports with experimental evidence, persistence was studied on dry surfaces using artificial contamination of a standardized type of surface in a laboratory. In most studies, bacteria were prepared in broth, water or saline. Viruses were usually prepared in a cell culture medium [48]. The main advantage is that the environmental conditions are consistent regarding temperature and air humidity. In addition, the effect of temperature or relative humidity can only be determined under controlled conditions, which are much easier to ensure in the laboratory. However, this may not always reflect the clinical situation, in which surfaces can be simultaneously contaminated with various nosocomial pathogens and different types of body fluids, secretions etc. Yet the question remains: what is the clinical evidence for the role of surfaces in nosocomial infections?

**Figure 1:**
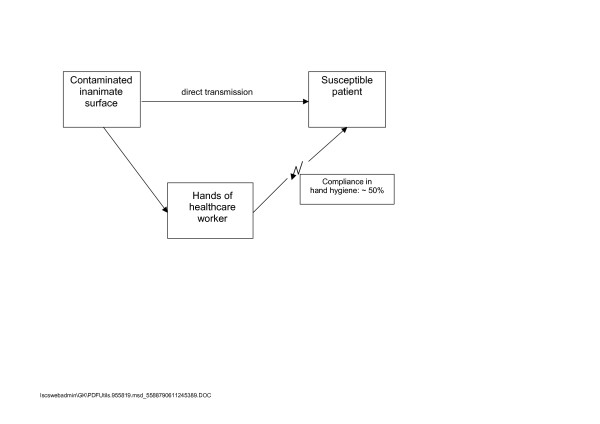
Common modes of transmission from inanimate surfaces to susceptible patients.

In hospitals, surfaces with hand contact are often contaminated with nosocomial pathogens [49-51], and may serve as vectors for cross transmission. A single hand contact with a contaminated surface results in a variable degree of pathogen transfer. Transmission to hands was most successful with *Escherichia coli*, *Salmonella *spp., *Staphylococcus aureus *(all 100%) [52], *Candida albicans *(90%) [53], *rhino *virus (61%) [54], *HAV *(22% – 33%) [55], and *rota *virus (16%) [56,57]. Contaminated hands can transfer viruses to 5 more surfaces [58] or 14 other subjects [59]. Contaminated hands can also be the source of re-contaminating the surface, as shown with *HAV *[55,58]. Compliance rates of healthcare workers in hand hygiene are known to be around 50% [7]. Due to the overwhelming evidence of low compliance with hand hygiene, the risk from contaminated surfaces cannot be overlooked (Figure 1).

The main route of transmission is via the transiently contaminated hands of the healthcare worker [60-62]. An outbreak of nosocomial infections due to *Acinetobacter baumannii *in a neurosurgical intensive care unit may serve as an example. A direct correlation was found between the number of environmental isolates obtained during screening and the number of patients who were colonized or infected with the same strain during the same calender month [63].

During outbreaks, the environment may play a significant role for transmission of nosocomial pathogens, as suggested by observational evidence. This has been described for various types of microorganisms, such as *Acinetobacter baumannii *[64-66], *Clostridium difficile *[67-69], MRSA [65,70], *Pseudomonas aeruginosa *[4,65], VRE [65,71-77], *SARS *[78,79], *rota- *[80,81], and *noro*virus [82]. However, the evidence to support a role of environmental contamination is not equally strong for all types of nosocomial pathogens. For *Clostridium difficile*, MRSA, and VRE, data are stronger than for other pathogens, such as *Pseudomonas aeruginosa *or *Acinetobacter baumannii*, of which multiple types were detected in the environment, and which did not always correlate with the acquired strain [83].

The role of surface disinfection for the control of nosocomial pathogens has been a contentious issue for some time [3]. Routine treatment of clean floors with various types of surface disinfectants (some of them had rather poor bactericidal activity) has been described to have no significant impact on the incidence of nosocomial infections [84]. Disinfection of surfaces in the immediate environment of patients, however, has been described to reduce acquisition of nosocomial pathogens such as VRE [85] or *Acinetobacter baumannii *[86]. It is therefore advisable to control the spread of nosocomial pathogens at least in the direct inanimate environment of the patient by routine surface disinfection.

## Conclusion

Most nosocomial pathogens can persist on inanimate surfaces for weeks or even months. Our review supports current guidelines which recommend a disinfection of surfaces in specific patient-care areas in order to reduce the risk of transmission of nosocomial pathogens from inanimate surfaces to susceptible patients.

## Competing interests

GK is a paid employee of Bode Chemie GmbH & Co. KG, Hamburg, Germany.

## Authors' contributions

All authors contributed to the conception, review of studies, and analysis of data. All authors were involved in drafting and revising the manuscript. All authors approved the final version of the manuscript.

**Table 3:** Persistence of clinically relevant viruses on dry inanimate surfaces.

Type of virus	Duration of persistence (range)	Source
Adenovirus	7 days – 3 months	[32, 34, 38–41, 111]
Astrovirus	7 – 90 days	[38]
Coronavirus	3 hours	[112, 113]
SARS associated virus	72 – 96 hours	[114]
Coxsackie virus	> 2 weeks	[34, 111]
Cytomegalovirus	8 hours	[115]
Echovirus	7 days	[39]
HAV	2 hours – 60 days	[35, 38, 41]
HBV	> 1 week	[116]
HIV	> 7 days	[117–119]
Herpes simplex virus, type 1 and 2	4.5 hours – 8 weeks	[34, 111, 118, 120]
Influenza virus	1 – 2 days	[39, 43, 121, 122]
Norovirus and feline calici virus (FCV)	8 hours – 7 days	[42, 45]
Papillomavirus 16	> 7 days	[123]
Papovavirus	8 days	[118]
Parvovirus	> 1 year	[118]
Poliovirus type 1	4 hours – < 8 days	[35, 118]
Poliovirus type 2	1 day – 8 weeks	[34, 38, 111]
Pseudorabies virus	≥ 7 days	[124]
Respiratory syncytial virus	up to 6 hours	[44]
Rhinovirus	2 hours – 7 days	[33, 125]
Rotavirus	6 – 60 days	[36 – 38, 41]
Vacciniavirus	3 weeks – > 20 weeks	[34, 126]

## Pre-publication history

The pre-publication history for this paper can be accessed here:



## References

[B1] Allerberger F, Ayliffe G, Bassetti M, Braveny I, Bucher A, Damani N, Daschner FD, Dettenkofer M, Ezpeleta C, Gastmeier P, Geffers C, Giamarellou H, Goldman D, Grzesiowski P, Gubina M, Haanen PE, Haydouchka I, Hubner J, Kalenic S, Van Knippenberg-Gordebeke G, Kranenburg AM, Krcmery V, Kropec A, Kruger W, Lemmen S, Mayhall CG, Meester M, Mehtar S, Munzinger J, Muzlovic I, Ojajarvi J, Rüden H, Scott G, Shah P, Tambic-Andraszevic A, Unertl K, Voss A, Weist K (2002). Routine surface disinfection in health care facilities: should we do it?. American Journal of Infection Control.

[B2] Rutala WA, Weber DJ (2001). Surface disinfection: should we do it?. Journal of Hospital Infection.

[B3] Cozad A, Jones RD (2003). Disinfection and the prevention of infectious disease. American Journal of Infection Control.

[B4] Engelhart S, Krizek L, Glasmacher A, Fischnaller E, Marklein G, Exner M (2002). Pseudomonas aeruginosa outbreak in a haematology-oncology unit associated with contaminated surface cleaning equipment. Journal of Hospital Infection.

[B5] Anonymous (2004). Anforderungen an die Hygiene bei der Reinigung und Desinfektion von Flächen. Bundesgesundheitsblatt.

[B6] Anonymous (2003). Guidelines for environmental infection control in health-care facilities. MMWR - Morbidity & Mortality Weekly Report.

[B7] Kampf G, Kramer A (2004). Epidemiologic background of hand hygiene and evaluation of the most important agents for scrubs and rubs. Clinical Microbiology Reviews.

[B8] Aitken C, Jeffries DJ (2001). Nosocomial spread of viral disease. Clinical Microbiology Reviews.

[B9] Neely AN, Maley MP (2000). Survival of enterococci and staphylococci on hospital fabric and plastic. Journal of Clinical Microbiology.

[B10] Wagenvoort JHT, Penders RJR (1997). Long-term in-vitro survival of an epidemic MRSA phage-group III-29 strain. Journal of Hospital Infection.

[B11] Rüden H, Gastmeier P, Daschner FD, Schumacher M (1997). Nosocomial and community-acquired infections in Germany. Summary of the results of the first national prevalence study (NIDEP). Infection.

[B12] Dickgiesser N (1978). Untersuchungen über das Verhalten grampositiver und gramnegativer Bakterien in trockenem und feuchtem Milieu. Zentralblatt für Bakteriologie und Hygiene, I Abt Orig B.

[B13] Hirai Y (1991). Survival of bacteria under dry conditions from a viewpoint of nosocomial infection. Journal of Hospital Infection.

[B14] Novak KD, Kowalski RP, Karenchak LM, Gordon YJ (1995). Chlamydia trachomatis can be transmitted by a nonporous plastic surface in vitro. Cornea.

[B15] Helke DM, Wong ACL (1994). Survival and growth characteristics of Listeria monocytogenes and Salmonella typhimurium on stainless steel and Buna-N rubber. Journal of Food Protection.

[B16] Gundermann KO (1972). Untersuchungen zur Lebensdauer von Bakterienstämmen im Staub unter dem Einfluß unterschiedlicher Luftfeuchtigkeit. Zentralblatt für Bakteriologie und Hygiene, I Abt Orig B.

[B17] Williams AP, Avery LM, Killham K, Jones DL (2005). Persistence of Escherichia coli O157 on farm surfaces under different environmental conditions. Journal of Applied Microbiology.

[B18] Jawad A, Snelling AM, Heritage J, Hawkey PM (1996). Influence of relative humidity and suspending menstrua on survival of Acinetobacter spp. on dry surfaces. Journal of Clinical Microbiology.

[B19] Jawad A, Snelling AM, Heritage J, Hawkey PM (1998). Exceptional desiccation tolerance of Acinetobacter radioresistens. Journal of Hospital Infection.

[B20] Noyce JO, Michels H, Keevil CW (2006). Potential use of copper surfaces to reduce survival of epidemic meticillin-resistant Staphylococcus aureus in the healthcare environment. Journal of Hospital Infection.

[B21] Augustine JL, Renshaw HW (1986). Survival of Corynebacterium pseudotuberculosis in axenic purulent exudate on common barnyard fomites. American Journal of Vetenary Research.

[B22] Wilks SA, Michels H, Keevil CW (2005). The survival of Escherichia coli O157 on a range of metal surfaces. International Journal of Food Microbiology.

[B23] Boehmler G, Gerwert J, Scupin E, Sinell HJ (1996). Zur Epidemiologie der Helicobacteriose des Menschen; Untersuchungen zur Überlebensfähigkeit des Erregers in Lebensmitteln. Deutsche Tierärztliche Wochenschrift.

[B24] Elmos T (1977). Survival of Neisseria gonorrhoeae on surfaces. Acta Dermato-Venereologica.

[B25] Wendt C, Dietze B, Dietz E, Rüden H (1997). Survival of Acinetobacter baumannii on dry surfaces. Journal of Clinical Microbiology.

[B26] Bale MJ, Bennett PM, Benninger JE, Hinton M (1993). The survival of bacteria exposed to dessication on surfaces associated with farm buildings. Journal of Applied Bacteriology.

[B27] Pérez JL, Gómez E, Sauca G (1990). Survival of gonococci from urethral discharge on fomites. European Journal of Clinical Microbiology and Infectious Diseases.

[B28] Neely AN (2000). A survey of gram-negative bacteria survival on hospital fabrics and plastics. Journal of Burn Care and Rehabilitation.

[B29] Webster C, Towner KJ, Humphreys H (2000). Survival of Acinetobacter on three clinically related inanimate surfaces. Infection Control and Hospital Epidemiology.

[B30] Smith CR (1942). Survival of tubercle bacilli: the viability of dried tubercle bacilli in unfiltered roomlight, in the dark, and in the refrigerator. American Review of Tuberculosis.

[B31] Blaschke-Hellmessen R, Kreuz M, Sprung M (1985). Umweltresistenz und natürliche Keimreservoire medizinisch bedeutsamer Sprosspilze. Zeitschrift für die gesamte Hygiene.

[B32] Hara J, Okomator S, Minekawa Y, Yamazaki K, Kase T (1990). Survival and disinfection of adenovirus type 19 and enterovirus 70 in ophthalmic practice. Japanese Journal of Ophthalmology.

[B33] Sattar SA, Karim YG, Springthorpe VS, Johnson-Lussenburg CM (1987). Survival of human rhinovirus type 14 dried onto nonporous inanimate surfaces: effect of relative humidity and suspending medium. Canadian Journal of Microbiology.

[B34] Mahl MC, Sadler C (1975). Virus survival on inanimate surfaces. Canadian Journal of Microbiology.

[B35] Mbithi JN, Springthorpe VS, Sattar SA (1991). Effect of relative humidity and air temperature on survival of hepatitis A virus on environmental surfaces. Applied and Environmental Microbiology.

[B36] Ansari SA, Springthorpe VS, Sattar SA (1991). Survival and vehicular spread of human rotaviruses: possible relation to seasonality of outbreaks. Reviews of Infectious Diseases.

[B37] Sattar S, Lloyd-Evans N, Springthorpe VS (1986). Institutional outbreaks of rotavirus diarrhoea: potential role of fomites and environmental surfaces as vehicles for virus transmission. Journal of Hygiene, Cambridge.

[B38] Abad FX, Villena C, Guix S, Caballero S, Pintó RM, Bosch A (2001). Potential role of fomites in the vesicular transmission of human astroviruses. Applied and Environmental Microbiology.

[B39] Wladowetz VW, Dmitrijewa RA, Safjulin AA (1974). Die Persistenz von Viren auf Oberflächen und die Anwendung der UV-Bestrahlung zur Virusdesinfektion. Zeitschrift für die gesamte Hygiene.

[B40] Gordon YJ, Gordon RY, Romanowski E, Araullo-Cruz TP (1993). Prolonged recovery of desiccated adenoviral serotypes 5, 8, and 19 from plastic and metal surfaces in vitro. Ophthalmology.

[B41] Abad FX, Pinto RM, Bosch A (1994). Survival of enteric viruses on environmental fomites. Applied and Environmental Microbiology.

[B42] d'Souza DH, Williams K, Jean J, Sair A, Jaykus L (2003). Persistence of Norwalk virus on environmental surfaces and its transfer to food: ; Washington, D.C...

[B43] Bean B, Moore BM, Sterner B, Peterson LR, Gerding DN, Balfour HH (1982). Survival of influenza viruses an environmental surfaces. The Journal of Infectious Diseases.

[B44] Hall CB, Douglas RG, Geiman JM (1980). Possible transmission by fomites of respiratory syncytial virus. The Journal of Infectious Diseases.

[B45] Clay S, Maherchandani S, Malik YS, Goyal SM (2006). Survival on uncommon fomites of feline calicivirus, a surrogate of noroviruses. American Journal of Infection Control.

[B46] Faix RG (1987). Comparative efficacy of handwashing agents against cytomegalievirus. Infection Control.

[B47] Weber DJ, Rutala WA (2001). The emerging nosocomial pathogens Cryptosporidium, Escherichia coli O157:h7, Helicobacter pylori, and hepatitis C: epidemiology, environmental survival, efficacy of disinfection, and control measures. Infection Control and Hospital Epidemiology.

[B48] Rzezutka A, Cook N (2004). Survival of human enteric viruses in the environment and food. FEMS Microbiology Reviews.

[B49] Bures S, Fishbain JT, Uyehara CF, Parker JM, Berg BW (2000). Computer keyboards and faucet handles as reservoirs of nosocomial pathogens in the intensive care unit. American Journal of Infection Control.

[B50] Catalano M, Quelle LS, Jeric PE, Di Martino A, Maimone SM (1999). Survival of Acinetobacter baumannii on bed rails during an outbreak and during sporadic cases. Journal of Hospital Infection.

[B51] Boyce JM, Potter-Bynoe G, Chenevert C, King T (1997). Environmental contamination due to methicillin-resistant Staphylococcus aureus: possible infection control implications. Infection Control and Hospital Epidemiology.

[B52] Scott E, Bloomfield SF (1990). The survival and transfer of microbial contamination via cloths, hands and utensils. Journal of Applied Bacteriology.

[B53] Rangel-Frausto MS, Houston AK, Bale MJ, Fu C, Wenzel RP (1994). An experimental model for study of Candida survival and transmission in human volunteers. European Journal of Clinical Microbiology and Infectious Diseases.

[B54] Gwaltney JM, Hendley JO (1982). Transmission of experimental rhinovirus infection by contaminated surfaces. American Journal of Epidemiology.

[B55] Mbithi JN, Springthorpe VS, Boulet JR, Sattar SA (1992). Survival of hepatitis A virus on human hands and its transfer on contact with animate an inanimate surfaces. Journal of Clinical Microbiology.

[B56] Ward RL, Bernstein DI, Knowlton DR, Sherwood JR, Young EC, Cusack TM, Rubino JR (1991). Prevention of surface-to-human transmission of rotaviruses by treatment with disinfectant spray. Journal of Clinical Microbiology.

[B57] Ansari SA, Sattar SA, Springthorpe VS, Wells GA, Tostawaryk W (1988). Rotavirus survival on human hands and transfer of infectious virus to inanimate and nonporous inanimate surfaces. Journal of Clinical Microbiology.

[B58] Barker J, Vipond IB, Bloomfield SF (2004). Effects of cleaning and disinfection in reducing the spread of norovirus contamination via environmental surfaces. Journal of Hospital Infection.

[B59] von Rheinbaben F, Schunemann S, Gross T, Wolff MH (2000). Transmission of viruses via contact in a household setting: experiments using bacteriophage strain phiXI174 as a model virus. Journal of Hospital Infection.

[B60] Laborde DJ, Weigle KA, Weber DJ, Kotch JB (1993). Effect of fecal contamination on diarrheal illness rates in day-care centers. American Journal of Epidemiology.

[B61] Mermel LA, Josephson SL, Dempsey J, Parenteau S, Perry C, Magill N (1997). Outbreak of Shigella sonnei in a clinical microbiology laboratory. Journal of Clinical Microbiology.

[B62] Farrington M, Ling J, Ling T, French GL (1990). Outbreaks of infection with methicillin-resistant Staphylococcus aureus on neonatal and burns units of a new hospital. Epidemiology and Infection.

[B63] Denton M, Wilcox MH, Parnell P, Green D, Keer V, Hawkey PM, Evans I, Murphy P (2004). Role of environmental cleaning in controlling an outbreak of Acinetobacter baumannii on a neurosurgical intensive care unit. Journal of Hospital Infection.

[B64] Fierobe L, Lucet JC, Decre D, Muller-Serieys C, Deleuze A, Joly-Guillou ML, Mantz J, Desmonts JM (2001). An outbreak of imipenem-resistant Acinetobacter baumannii in critically ill surgical patients. Infection Control and Hospital Epidemiology.

[B65] Lemmen SW, Häfner H, Zolldann D, Stanzel S, Lütticken R (2004). Distribution of multi-resistant Gram-negative versus Gram-positive bacteria in the hospital inanimate environment. Journal of Hospital Infection.

[B66] Ling ML, Ang A, Wee M, Wang GC (2001). A nosocomial outbreak of multiresistant Acinetobacter baumannii originating from an intensive care unit. Infection Control and Hospital Epidemiology.

[B67] Hanna H, Raad I, Gonzalez V, Umphrey J, Tarrand J, Neumann J, Champlin R (2000). Control of nosocomial Clostridium difficile transmission in bone marrow transplant patients. Infection Control and Hospital Epidemiology.

[B68] Verity P, Wilcox MH, Fawley W, Parnell P (2001). Prospective evaluation of environmental contamination by Clostridium difficile in isolation side rooms. Journal of Hospital Infection.

[B69] Kaatz GW, Gitlin SD, Schaberg DR, Wilson KH, Kauffman CA, Seo SM, Fekety R (1988). Acquisition of Clostridium difficile from the hospital environment. American Journal of Epidemiology.

[B70] Fitzpatrick F, Murphy OM, Brady A, Prout S, Fenelon LE (2000). A purpose built MRSA cohort study. Journal of Hospital Infection.

[B71] Falk PS, Winnike J, Woodmansee C, Desai M, Mayhall CG (2000). Outbreak of vancomycin-resistant enterococci in a burn unit. Infection Control and Hospital Epidemiology.

[B72] Gray JW, George RH (2000). Experience of vancomycin-resistant enterococci in a children's hospital. Journal of Hospital Infection.

[B73] McCarthy KM, Van Nierop W, Duse A, Von Gottberg A, Kassel M, Perovic O, Smego R (2000). Control of an outbreak of vancomycin-resistant Enterococcus faecium in an oncology ward in South Africa: effective use of limited resources. Journal of Hospital Infection.

[B74] Hanna H, Umphrey J, Tarrand J, Mendoza M, Raad I (2001). Management of an outbreak of vancomycin-resistant enterococci in the medical intensive care unit of a cancer center. Infection Control and Hospital Epidemiology.

[B75] Martinez JA, Ruthazer R, Hansjosten K, Barefoot L, Snydman DR (2003). Role of environmental contamination as a risk factor for acquisition of vancomycin-resistant enterococci in patients treated in a medical intensive care unit. Archives of Internal Medicine.

[B76] Bonten MJM, Hayden MK, Nathan C, van Voorhis J, Matushek M, Slaughter S, Rice T, Weinstein RA (1996). Epidemiology of colonization of patients and environment with vancomycin-resistant enterococci. The Lancet.

[B77] Duckro AN, Blom DW, Lyle EA, Weinstein RA, Hayden MK (2005). Transfer of vancomycin-resistant enterococci via health care worker hands. Archives of Internal Medicine.

[B78] Mukhopadhyay A, Tambyah PA, Singh KS, Lim TK, Lee KH (2004). SARS in a hospital visitor and her intensivist. Journal of Hospital Infection.

[B79] Chen YC, Huang LM, Chan CC, Su CP, Chang SC, Chang YY, Chen ML, Hung CC, Chen WJ, Lin FY, Lee YT (2004). SARS in hospital emergency room. Emerging Infectious Diseases.

[B80] Butz AM, Fosarelli P, Dick J, Cusack T, Yolken R (1993). Prevalence of rotavirus on high-risk fomites in day-care facilities. Pediatrics.

[B81] Wilde J, Van R, Pickering L, Eiden J, Yolken R (1992). Detection of rotaviruses in the day care environment by reverse transcriptase polymerase chain reaction. The Journal of Infectious Diseases.

[B82] Chadwick PR, Beards G, Brown D, Caul EO, Cheesbrough J, Clarke I, Curry A, O'Brien S, Quigley K, Sellwood J, Westmoreland D (2000). Management of hospital outbreaks of gastro-enteritis due to small roundstructured viruses. Journal of Hospital Infection.

[B83] Hota B (2004). Contamination, disinfection, and cross-colonization: are hospital surfaces reservoirs for nosocomial infection?. Clinical Infectious Diseases.

[B84] Dharan S, Mourouga P, Copin P, Bessmer G, Tschanz B, Pittet D (1999). Routine disinfection of patients' environmental surfaces. Myth or reality?. Journal of Hospital Infection.

[B85] Hayden MK, Bonten MJM, Blom DW, Lyle EA, van de Vijver DAMC, Weinstein RA (2006). Reduction in acquisition of vancomycin-resistant enterococcus after enforcement of routine environmental cleaning measures. Clinical Infectious Diseases.

[B86] Wilks M, Wilson A, Warwick S, Price E, Kennedy D, Ely A, Millar MR (2006). Control of an outbreak of multidrug-resistant Acinetobacter baumannii-calcoaceticus colonization and infection in an intensive care unit (ICU) without closing the ICU or placing patients in isolation. Infection Control and Hospital Epidemiology.

[B87] Musa EK, Desai N, Casewell MW (1990). The survival of Acinetobacter calcoaceticus inoculated on fingertips and on formica. Journal of Hospital Infection.

[B88] Getchell-White SI, Donowitz LG, Groschel DH (1989). The inanimate environment of an intensive care unit as a potential source of nosocomial bacteria: evidence for long survival of Acinetobacter calcoaceticus. Infection Control and Hospital Epidemiology.

[B89] Hahn H, Arvand M, Hahn H, Falke D, Kaufmann SHE and Ullmann U (2001). Bordetellen. Medizinische Mikrobiologie und Infektiologie.

[B90] Mitscherlich E, Marth EH (1984). Microbial survival in the environment.

[B91] Boucher SN, Chamberlain AHL, Adams MR (1998). Enhanced survival of Campylobacter jejuni in association with wood. Journal of Food Protection.

[B92] Kim KH, Fekety R, Batts DH, Brown D, Cudmore M, Silva J, Waters D (1981). Isolation of Clostridium difficile from the environment and contacts of patients with antibiotic-associated colitis. The Journal of Infectious Diseases.

[B93] Mulligan ME, George WL, Rolfe RD, Finegold SM (1980). Epidemiologic aspects of Clostridium difficile-induced diarrhea and colitis. The American Journal of Clinical Nutrition.

[B94] McFarland LV, Stamm WE (1986). Review of Clostridium difficile-associated diseases. American Journal of Infection Control.

[B95] Falsey AR, Walsh EE (1993). Transmission of Chlamydia pneumoniae. The Journal of Infectious Diseases.

[B96] Crosbie WE, Wright HD (1941). Diphtheria bacilli in floor dust. The Lancet.

[B97] Maule A (2000). Survival of verocytotoxigenic Escherichia coli O157 in soil, water and on surfaces. Symposium Series (Society for Applied Microbiology).

[B98] Abrishami SH, Tall BD, Bruursema TJ, Epstein PS, Shah DB (1994). Bacterial adherence and viability on cutting board surfaces. Journal of Food Safety.

[B99] Kampf G, Dietze B, Große-Siestrup C, Wendt C, Martiny H (1998). The microbiocidal action of a new silver-containing polymer (SPI-ARGENT II). Antimicrobial Agents and Chemotherapy.

[B100] Noskin GA, Stosor V, Cooper I, Peterson LR (1995). Recovery of vancomycin-resistant enterococci on fingertips and environmental surfaces. Infection Control and Hospital Epidemiology.

[B101] Wendt C, Wiesenthal B, Dietz E, Rüden H (1998). Survival of vancomycin-resistant and vancomycin-susceptible enterococci on dry surfaces. Journal of Clinical Microbiology.

[B102] Mielke M, Hahn H, Hahn H, Falke D, Kaufmann SHE and Ullmann U (2001). Anthropozoonoseerreger ohne Familienzugehörigkeit: Listerien, Brucellen, Francisellen und Erysipelothrix. Medizinische Mikrobiologie und Infektiologie.

[B103] Gould JC, Williams REO and Shooters RA (1963). Pseudomonas pyocyanea infections in hospitals. Infection in hospitals.

[B104] Panagea S, Winstanley C, Walshaw MJ, Ledson MJ, Hart CA (2005). Environmental contamination with an epidemic strain of Pseudomonas aeruginosa in a Liverpool cystic fibrosis centre, and study of its survival on dry surfaces. Journal of Hospital Infection.

[B105] Robertson MH (1972). Survival of S. typhimurium in floor dust: a possible reservoir of infection in institutions. Public Health.

[B106] Nass W (1977). Zur Überlebensdauer von Shigellen auf Plastikwerkstoffen. Zeitschrift für die gesamte Hygiene.

[B107] Islam MS, Hossain MA, Khan SI, Khan MN, Sack RB, Albert MJ, Huq A, Colwell RR (2001). Survival of Shigella dysenteriae type 1 on fomites. Journal of Health, Population, and Nutrition.

[B108] Wagenvoort JH, Sluijsman W, Penders RJ (2000). Better environmental survival of outbreak vs. sporadic MRSA isolates. Journal of Hospital Infection.

[B109] Barua D (1970). Survival of cholera vibrios in food, water and fomites. Public Health Papers.

[B110] Traore O, Springthorpe VS, Sattar SA (2002). A quantitative study of the survival of two species of Candida on porous and non-porous environmental surfaces and hands. Journal of Applied Microbiology.

[B111] Gerth HJ, Thofern E and Botzenhart K (1983). Viren und virale Erkrankungen. Hygiene und Infektionen im Krankenhaus.

[B112] Sizun J, Yu MW, Talbot PJ (2000). Survival of human coronaviruses 229E and OC43 in suspension and after drying on surfaces: a possible source of hospital-acquired infections. Journal of Hospital Infection.

[B113] Gagneur A, Legrand MC, Picard B, Baron R, Talbot PJ, de Parscau L, Sizun J (2002). Nosocomial infections due to human coronaviruses in the newborn. Archives of Pediatrics.

[B114] Duan SM, Zhao XS, Wen RF, Huang JJ, Pi GH, Zhang SX, Han J, Bi SL, Ruan L, Dong XP (2003). Stability of SARS coronavirus in human specimens and environment and its sensitivity to heating and UV irradiation. Biomedical and Environmental Sciences.

[B115] Roger G, Faix MD (1985). Survival of cytomegalievirus on environmental surfaces. The Journal of Pediatrics.

[B116] Bond WW, Favero MS, Petersen NJ, Gravelle CR, Ebert JW, Maynhard JE (1981). Survival of hepatitis B virus after drying and storage for one week. The Lancet.

[B117] Barré-Sinoussi F, Nugeyre MT, Chermann JC (1985). Resistance of AIDS virus at room temperature. The Lancet.

[B118] von Rheinbaben F, Wolff MH (2002). Handbuch der Virusdesinfektion.

[B119] Tjotta E, Hungnes O, Grinde B (1991). Survival of HIV-1: activity after disinfection, temperature and pH changes, or drying. Journal of Medical Virology.

[B120] Nerurkar LS, West F, May M, Madden DL, Sever JL (1983). Survival of herpes simplex virus in water specimens collected from hot spa facilities and on plastic surfaces. The Journal of the American Medical Association.

[B121] Brady MT, Evans J, Cuartas J (1990). Survival and disinfection of parainfluenza viruses on environmental surfaces. American Journal of Infection Control.

[B122] Pirtle EC, Beran GW (1991). Virus survival in the environment. Revue Scientifique et Technique.

[B123] Roden RBS, Lowy DR, Schiller JT (1997). Papillomavirus is resistant to desiccation. The Journal of Infectious Diseases.

[B124] Schoenbaum MA, Freund JD, Beran GW (1991). Survival of pseudorabies virus in the presence of selected diluents and fomites. Journal of the American Veterinary Medical Association.

[B125] Reed S (1975). An investigation of the possible transmission of rhinovirus colds through direct contact. Journal of Hygiene, London.

[B126] Mahnel H (1987). Experimentelle Ergebnisse über die Stabilität von Pockenviren unter Labor- und Umweltbedingungen. Zentralblatt für Veterinärmedizin.

